# Extracorporeal membrane oxygenation for paediatric refractory hypoxic respiratory failure caused by adenovirus in Shanghai: a case series

**DOI:** 10.1186/s12887-022-03197-2

**Published:** 2022-03-16

**Authors:** Yun Cui, Jingyi Shi, Yiping Zhou, Jiaying Dou, Xi Xiong, Ting Sun, Yijun Shan, Tingting Xu, Ye Lu, Yucai Zhang

**Affiliations:** Department of Critical Care Medicine, Shanghai Children’s Hospital, Shanghai Jiao Tong University School of Medicine, No.355 Luding Road, Putuo District, Shanghai, 200062 China

**Keywords:** Refractory hypoxemia, Adenovirus, Extracorporeal membrane oxygenation, Survival, Child

## Abstract

**Background:**

To assess the outcome of extracorporeal membrane oxygenation (ECMO) for severe adenovirus (Adv) pneumonia with refractory hypoxic respiratory failure (RHRF) in paediatric patients.

**Methods:**

A retrospective observational study was performed in a tertiary paediatric intensive care unit (PICU) in China. Patients with RHRF caused by Adv pneumonia who received ECMO support after mechanical ventilation failed to achieve adequate oxygenation between 2017 and 2020 were included. The outcome variables were the in-hospital survival rate and the effects of ECMO on the survival rate.

**Results:**

In total, 18 children with RHRF received ECMO. The median age was 19 (9.5, 39.8) months, and the median ECMO duration was 196 (152, 309) h. The in-hospital survival rate was 72.2% (13/18). Thirteen patients (72.2%) required continuous renal replacement therapy (CRRT) due to fluid imbalance or acute kidney injury (AKI). At ECMO initiation, compared with survivors, nonsurvivors had a lower PaO_2_/FiO_2_ ratio [49 (34.5, 62) vs. 63 (56, 71); *p* = 0.04], higher oxygen index (OI) [41 (34.5, 62) vs. 30 (26.5, 35); *p* = 0.03], higher vasoactive inotropic score (VIS) [30 (16.3, 80) vs. 100 (60, 142.5); *p* = 0.04], longer duration from mechanical ventilation to ECMO support [8 (4, 14) vs. 4 (3, 5.5) h, *p*=0.02], and longer time from confirmed RHRF to ECMO initiation [9 (4.8, 13) vs. 5 (1.3, 5.5) h; *p* = 0.004]. Patients with PaO_2_/FiO_2_ <61 mmHg or an OI >43 and hypoxic respiratory failure for more than 9 days before the initiation of ECMO had worse outcomes.

**Conclusions:**

ECMO seemed to be effective, as severe paediatric Adv pneumonia patients with RHRF had a cumulative survival rate of 72.2% in our study. Our study provides insight into ECMO rescue in children with severe Adv pneumonia.

**Supplementary Information:**

The online version contains supplementary material available at 10.1186/s12887-022-03197-2.

## Background

Adenovirus (Adv) may cause rapidly progressive, life-threatening illness with multiple organ involvement in both immunocompetent and immunocompromised individuals. Patients with severe Adv infection develop refractory hypoxemia and/or acute respiratory distress syndrome (ARDS) [1, 2]. Extracorporeal membrane oxygenation (ECMO) has been used as a salvage therapy in severe Adv patients with ARDS and refractory hypoxic respiratory failure (RHRF) who respond poorly to conventional therapy [3–5]. In 2014, Prodhan and colleagues reviewed the Extracorporeal Life Support Organization (ELSO) registry from 1998 to 2009 and found that the hospital survival rate was 38% (62/163) in children requiring ECMO support for Adv infection. Among neonates (<31 days of age), the survival at hospital discharge was only 11% (6/54) [5]. Recently, Ramanathan et al. [6] analysed 25 years of ELSO registry data of those in all age groups who needed ECMO due to severe Adv pneumonia. The in-hospital mortality rate was 58%, with no significant improvement from 1992 to 2016. This outcome strongly indicated that ECMO as rescue therapy was not satisfactory.

In recent years, outbreaks of Adv infection in children have occurred in China [7, 8]. In 2019, the China Health and Health Commission issued expert recommendations for the prevention and treatment of Adv pneumonia in children (http://www.nhc.gov.cn/yzygj/s7653p/201906/ab8ec27548ea48f793734e8d09c8d42c/files/68cc1dca88f34d5692a40347d31b4852.pdf). This retrospective case series study was undertaken to describe our experience with cases of severe Adv pneumonia with RHRF requiring ECMO from January 2017 to August 2020 in a paediatric intensive care unit (PICU) in Shanghai.

## Methods

### Patients and diagnosis criteria

**Figure 1:**
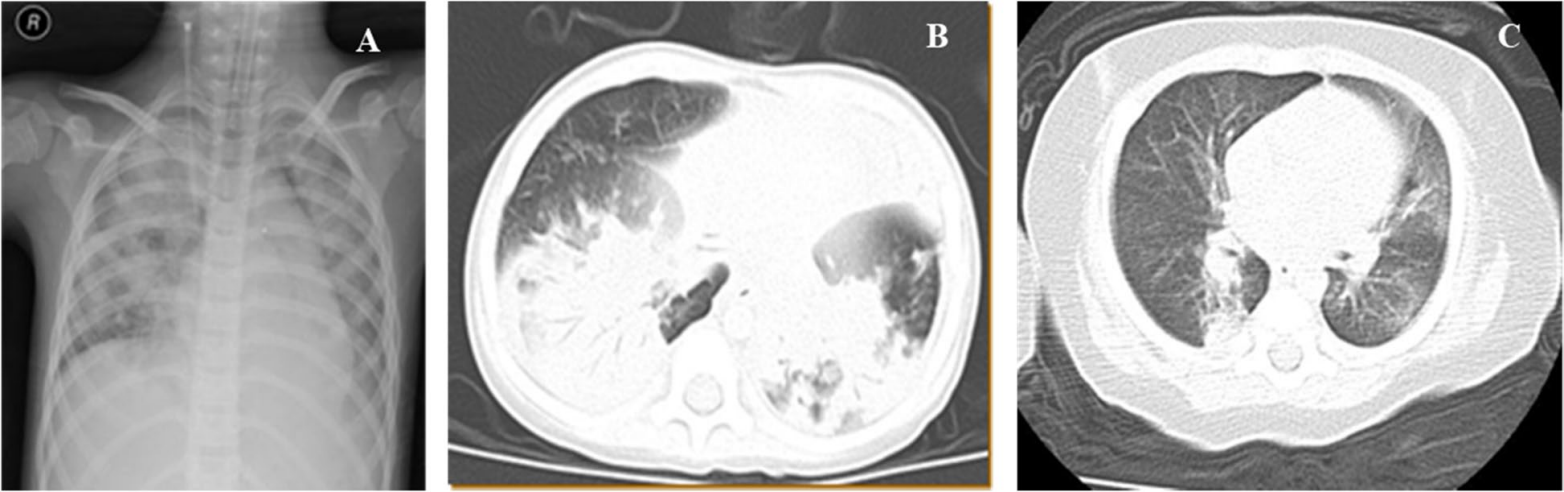
**Fig. 1** Radiologic characteristic of Adv pneumonia. **A **X-ray showed bilateral pulmonary effusion. **B** Pulmonary CT showed lung consolidation. **C** Bronchial dilatation and transparent lung

Children with Adv pneumonia complicated with respiratory failure were admitted to the PICU (36 beds in a tertiary university hospital) of Shanghai Children’s Hospital affiliated to Shanghai Jiao Tong University School of Medicine. Adv pneumonia was diagnosed according to the following criteria: 1) symptoms of acute lower respiratory tract illness; 2) lung infiltration on chest radiography or computed tomography (CT) (Fig. 1); and 3) detection of Adv DNA from bronchoalveolar lavage (BAL) fluid by RT–PCR or by metagenomic next-generation sequencing (mNGS). ARDS was diagnosed using the Berlin criteria [9], and RHRF was defined as an arterial oxygen partial pressure to fractional inspired oxygen ratio (PaO_2_/FiO_2_) of <80 mmHg, with no improvement with protective mechanical ventilation. This study was approved by the Ethics Committee of Shanghai Children’s Hospital affiliated to Shanghai Jiao Tong University (2016R007-F01, 2018R52-F01, and 2020R032-F01). Informed consent was obtained from parents of all study participants. Patient records and information were anonymized and deidentified before analysis.

### Indications for ECMO

Our ECMO team made all decisions regarding treatment with ECMO. If RHRF was the only indication, venovenous (VV)-ECMO was carried out. In patients with acute cardiovascular deterioration, venoarterial (VA)-ECMO was carried out. The requirement for VV-ECMO was based on the following: 1) severe hypoxemia with a PaO_2_/FiO_2_ of < 50 mmHg for > 3 h or < 80 mmHg for > 6 h or pH < 7.25 and a partial pressure of arterial CO_2_ of ≥60 mmHg for > 6 h [10]; and 2) femoral vessels ≥3 mm for venous drainage or return in children weighing approximately 15 kg or aged 2 years (select multicannulation VV-ECMO mode due to lack of double-lumen cannulas in China).

VA-ECMO was applied when RHRF patients had persistent haemodynamic instability or worsening cardiac dysfunction, including 1) a cardiac index (CI) less than 2.0 L/min.m^2^; or 2) circulatory dysfunction with persistent lactatemia (LA) greater than 4 mmol/L and a vasoactive inotropic score (VIS) greater than 50. The VIS was calculated as ([(epinephrine+ norepinephrine) ug/kg.min] × 100 + [(dobutamine + dopamine) ug/kg.min] + [milrinone ug/kg.min] × 15. Intravenous neuromuscular blockade was started if the peak inspiratory pressure was approximately 28-30 cmH_2_O and the patient was hypoxemic and continued to show laboured breathing despite adequate sedation [11]. Our ECMO unit has been a middle-volume registered ELSO member (No. 663) since 2018.

### ECMO procedures

ECMO was performed using a Centrifugal Rotaflow Pump® (MEDOS HILITE 2400LT, Medizintechnik AG, and the Maquet Quadrox PLS diffusion membrane oxygenator) and 8-17Fr venous and 8-15Fr arterial cannulas (Medtronic or Edward’s Lifesciences, Irvine, CA, USA). VA-ECMO supports cardiopulmonary failure, whereas VV-ECMO supports lung failure without severe cardiovascular dysfunction. Cannulation was guided by ultrasound imaging. For VV-ECMO, cannulation was carried out in the right internal jugular vein and the femoral vein. For VA-ECMO, cannulation was carried out in the jugular/femoral artery and vein.

For anticoagulation, a bolus dose of 50-100 U/kg unfractionated heparin was administered at the time of cannulation, followed by 20-50 U/kg/hr continuous infusion; the dosage was adjusted to maintain an activated coagulation time (ACT) of 180-220 s or kaolin partial thromboplastin time 1.5 or 2 times that of normal arterial blood.

### Data collection

The clinical course of each patient was obtained through a computerized medical record database in the PICU. Collected parameters included age, sex, paediatric risk of mortality III (PRISM III) score, PaO_2_/FiO_2_, PaCO_2_, CI, mean arterial pressure (MAP), comorbidities, and hospital-related infectious pathogens, as well as biochemical indicators of organ function (total bilirubin [TBIL]; lactic acid [LA]; serum creatinine [sCr];, etc.). The primary outcome was the hospital survival rate, and secondary outcomes included the length of PICU or hospital stay and ECMO-associated complications.

### Statistical analysis

All statistical analyses were performed with IBM SPSS Statistics V.22.0 (SPSS Inc, Chicago). Continuous variables were summarized as means± standard deviations (SDs) for normally distributed data and as medians (interquartile ranges, IQRs) for nonnormally distributed data. Independent-samples *t* tests (for normally distributed data), Mann–Whitney *U* tests (for nonnormally distributed data), or *chi*-square tests (for categorical variables) were used to compare parameters between the two groups. A *P* value less than 0.05 was considered statistically significant.

## Results

In total, 88 patients with severe Adv pneumonia were admitted to the PICU from January 2017 to August 2020. Among them, 18 children with Adv type 7 (Adv-7) pneumonia complicated by RHRF and mechanical ventilation treatment failure were included in this series. Eleven (61.1%) patients were diagnosed with ARDS. The median age was 19 (9.5, 39.8) months, and 9 patients were male. The baseline pre-ECMO demographics are presented in Tables 1 and 2. The radiologic characteristics of Adv pneumonia are shown in Fig. 1.

**Table 1:** Clinical details of pediatric patients in extracorporeal membrane oxygenation cohort (*n*=18)

Patient	Time	Clinical Synopsis	PRISM III Score	Hours on ECMO	ICU Length of Stay (d)	Days on Mechanical Ventilation	Interval time from onset to ECMO initiation (d)	Time from confirmed respiratory failure to ECMO support (d)	Clinical outcome
1	January 2017	21-month-old girl, fever and cough for 10 days, tachypnea for 3 days	14	237	31	21	23	5	Survive
2	July 2019	8-years-old girl, coughed and fever for 10 days, tachypnea and for 1 day with lethargy	9	173	17	17	26	7	Non survive
3	September 2017	7-month-old boy, wheezed and fever for one week, exhausted for 1 day	15	122	21	14	19	5	Survive
4	April 2017	9- month-old girl, coughed and fever for 1 week, tachypnea, cyanosis and lethargy for 1 day	12	332	50	27	10	1	Survive
5	January 2018	11-month-old girl, fever for 6 days, cough for 5 days and pale complexion for 1 day	15	330	63	63	20	10	Survive
6	July 2017	7-month-old boy, coughed and wheeze for 6 days, tachypnea for 1 day	11	505	35	35	20	14	Non survive
7	February 2019	19-month-old boy, cough for 6 days with fiver, lethargy for 1 day	17	244	15	15	10	4	Non survive
8	May 2019	9-month-old boy, fever and cough for 6 days, tachypnea and cyanosis for 1 day	13	451	23	24	10	3	Non survive
9	April 2019	13-month-old girl, cough for 10 days, fever for 6 days, tachypnea with lethargy for 1 day	13	302	58	40	15	5	Survive
10	May 2019	16-month-old girl, cough with fever for 10 days, tachypnea for 1 day, cyanosis and lethargy for 2 hours	12	166	28	16	21	6	Survive
11	January 2019	3-years-old girl, fever for 9 days, cough for 7 days, tachypnea for 1 day	2	159	26	10	10	2	Survive
12	September 2019	2-years-old boy, coughed and fever for 10 days, tachypnea for 4 days, lethargy and cyanosis for 4 hours	9	135	27	17	10	5	Survive
13	October 2019	3-years-old boy, fever for 8 days, cough for 5 days, cyanosis for 6 hours	4	158	39	12	7	3	Survive
14	November 2019	6-years-old boy, fever for 6 days, wheeze for 4 days, tachypnea and lethargy for 1 day	7	219	208	18	5	9	Survive
15	January 2020	2-year-old boy, coughed with fever for 16 days, tachypnea for 2 days	7	92	17	8	26	5	Survive
16	July 2020	7-month-old girl, coughed and wheezed for 6 days, fever for 2 days, tachypnea for 1 day	8	170	18	13	9	3	Survive
17	July 2020	4-years-old girl, coughed for 6 days with fever, tachypnea for 1 day and cyanosis for 3 hours	22	122	15	13	6	2	Survive
18	August 2020	19-month-old boy, coughed and wheezed for 10 days, fever for 5 days, tachypnea for 1 day	16	240	33	33	16	12	Non survive

**Table 2:** baseline characteristics, management, and outcome of 18 patients treated with ECMO for Adv pneumonia caused RHRF

	Total *N*=18 Median (IQR), or N (%)	Survival *N*=13	Non survival *N*=5	*P*
Baseline characteristics				
Age, mo	19 (9.5, 39.8)	21 (10.3, 40.5)	19 (8.5, 59.5)	0.76
Male sex	9 (50)	5 (38.5)	4 (80)	0.11
On admission				
PRISM III	12 (7.8, 15)	12 (7, 14.5)	13 (10, 16.5)	0.36
Before ECMO				
Cardiovascular dysfunction	14 (77.8)	9 (69.2)	5 (100)	0.16
Corticosteroid therapy	18 (100)	13 (100)	5 (100)	
vasoactive inotropic score (VIS)	33.8 (19.4, 105)	30 (16.3, 80)	100 (60, 142.5)	0.04*
Organ function				
MAP, mmHg	59.5 (51.3, 65.5)	60 (55, 66)	55 (47, 65)	0.39
CI, L/min/m^2^	3.2 (2.9, 3.6)	3.4 (3.1, 3.7)	2.8 (2.5, 3.2)	0.05
TBIL, mmoL/L	15.7 (9.9, 33.2)	14.1 (6.7, 20.2)	45.2 (27.5, 112.1)	0.008*
sCr, umol/L	27 (18.8, 36.5)	26 (14.5, 31)	36 (20.5, 58)	0.09
Rescue therapy				
Prone position ventilation	16 (88.9)	11 (84.6)	5 (100)	0.35
CRRT	13 (72.2)	8 (61.5)	5 (100)	0.23
Inhaled nitric oxide	5 (27.8)	4 (30.8)	1 (20)	0.65
Time from onset to ECMO, d	15.5 (9.8, 20.3)	15 (9.5, 20.5)	19 (8, 23)	0.69
Time from PICU admission to ECMO, d	5 (3, 7.5)	5 (3, 5.5)	7 (3.5, 13)	0.09
Time from intubation to ECMO, d	4.5 (3, 7.3)	4 (3, 5.5)	8 (4, 14)	0.02*
Time from confirmed RHRF to ECMO, d	5 (1.9, 6.6)	5 (1.3, 5.5)	9 (4.8, 13)	0.004*
Tidal volume, ml/kg	6.9 (5.9, 7,5)	7 (6.8, 7.6)	5.5 (4.9, 6.1)	<0.001*
PEEP, cmH_2_O	10 (8, 12)	9 (8, 11)	11 (10, 14.5)	0.025*
Plateau pressure, cmH_2_O	19 (17.8, 20.3)	18 (17, 20.5)	19 (19, 22.5)	0.33
Peak inspiratory pressure, cmH_2_O	31 (27.8, 35)	28 (26.5, 33)	35 (30, 36)	0.1
Oxygenation index (OI)	33.5 (27.8, 41.5)	30 (26.5, 35)	41 (34.5, 62)	0.03*
PaO_2_/FiO_2_, mmHg	60 (50.5, 67.8)	63 (56, 71)	49 (40, 57)	0.04*
SaO_2_, %	78.5 (75.3, 87)	81 (78, 87.5)	73 (69.5, 77)	0.04*
Arterial, pH	7.41 (7.31, 7.49)	7.37 (7.31, 7.49)	7.49 (7.38, 7.58)	0.09
PaCO_2_, mmHg	60 (44, 78)	62 (48.6, 83.5)	58 (38.5, 78)	0.51
Arterial lactate, mmol/L	2.3 (1.5, 4.6)	2 (0.9, 3.9)	3.7 (2.7, 5.2)	0.08
Immune status, %				
CD3^+^	48.8 (38.7, 57.8)	51.8 (38.4, 60.1)	42.8 (36.2, 62.9)	0.73
CD4^+^	29.8 (21.5, 38.6)	28.6 (18.6, 34.2)	39.2 (31.1, 47.6)	0.02*
CD8^+^	20.6 (16.3, 26.4)	18.7 (13.7, 27.2)	22.2 (17.9, 33.9)	0.63
CD19^+^	40.8 (28.2, 52.4)	39.4 (23.3, 53.3)	42.1 (28.2, 53.9)	0.82
NK	4 (1.7, 4.6)	4.1 (3.3, 4.8)	0.7 (0.2, 3.1)	0.04*
Complications and outcome				
Nosocomial infection, n	8 (44.4)	4 (30.8)	4 (80)	0.05*
ARDS, n	11 (61.1)	6 (46.2)	5 (100)	0.03*
Liver dysfunction, n	12 (66.7)	7 (53.8)	5 (100)	0.06
AKI, n	7 (38.9)	2 (15.4)	5 (100)	0.001*
Cardiovascular dysfunction, n	13 (72.2)	8 (61.5)	5 (100)	0.10
GI dysfunction, n	9 (50)	5 (38.5)	4 (80)	0.11
Encephalopathy, n	3 (16.7)	1 (7.7)	2 (40)	0.09
Length of ECMO, h	196 (152, 309)	166 (128.5, 269.5)	244 (206.5, 478)	0.03*
Length of MV, d	16 (13, 27.8)	15 (12.5, 29.5)	20 (15, 28.5)	0.99
Length of PICU stay, d	27.5 (17.8, 41.8)	27 (19.5, 54)	23 (16, 33)	0.31
Length of hospital stay, d	35 (27.3, 58)	48 (31.5, 71.5)	28 (19, 34)	0.15
V-V ECMO, n	5 (27.8)	3 (23.1)	3 (60)	0.14
Mortality	5 (27.8)			

### Radiologic characteristic of Adv pneumonia

X-ray showing bilateral pulmonary effusion (A). Pulmonary CT showing lung consolidation (B), bronchial dilatation and lung transparency (C). Capillary leakage syndrome occurred in 6 patients and resulted in fluid overload.

The median interval times from onset and PICU admission to ECMO initiation were 15.5 (9.8, 20.3) days and 5 (3,7.5) days, respectively. Eleven patients underwent VA-ECMO, five underwent VV-ECMO, 1 underwent VV-to-VA conversion, and 1 underwent VA-to-VV conversion. The PaO_2_/FiO_2_ was 54 mmHg (IQR 50.5, 60.5) for those receiving VV-ECMO and 65 mmHg (IQR 51, 72) for those receiving VA-ECMO (*p*=0.31) at initiation. Nonsurvivors had a longer duration from intubation to ECMO support [8 (4, 14) vs. 4 (3, 5.5) h, *p*=0.02] and a longer time from confirmed RHRF to ECMO support [9 (4.8, 13) vs. 5 (1.3, 5.5) h; *p* = 0.004] than survivors. The median blood flow at ECMO initiation and at 24 h was 85 ml/kg.min (IQR, 80, 95) and 75 ml/kg.min (IQR 62, 90), respectively. Average blood flow rates at the beginning of ECMO and at 24 h, 48 h and 7 days of ECMO support are shown in Table 3.

**Table 3:** The ECMO setting parameters, ventilator tidal volume and Cdyn in patients receiving ECMO

Case	ECMO mode	Outcome	*Blood flow, mL/kg/min*				***ECMOFiO2***				***Sweep gas flow, L/min***				***Vt, ml/kg***				***Cdyn, ml/cmH***_***2***_***O***			
			D0	24hr	48h	D7	D0	24hr	48h	D7	D0	24hr	48h	D7	D0	24hr	48h	D7	D0	24hr	48h	D7
**1**	VA	Survival	73	70	44	53	100	100	45	40	2.5	2.5	2.5	1.5	4.5	5	6	7.8	0.34	0.4	0.3	0.4
**2**	VV-VA	Dead	95	95	68	114	100	100	100	100	3	2	2.2	3	3.8	3.5	3.3	2.5	0.25	0.3	0.25	0.1
**3**	VA	Survival	87	80	53	/	100	100	70	/	2	1.5	1.5	/	4.5	6	9	8.1	0.34	0.3	0.55	0.5
**4**	VA	Survival	80	50	42	63	100	100	100	100	1	1	1	1	4.5	5	6.5	6.8	0.31	0.32	0.12	0.24
**5**	VA	Survival	100	100	92	53	100	100	100	60	2	2	2	2.5	4.1	4.7	5.7	7.5	0.31	0.5	0.52	0.56
**6**	VA	Dead	85	60	75	70	100	100	100	100	1.5	1.2	1.5	1.6	3.5	4.6	3.5	2.5	0.54	0.3	0.32	0.23
**7**	VA-VV	Dead	95	92	100	150	100	100	100	100	2	3	3	3	3.6	2.7	2.5	3.3	0.37	0.5	0.4	0.08
**8**	VV	Dead	120	120	77	72	100	100	100	60	1	1.5	0.8	0.6	4.4	6.5	8	6.9	0.37	0.3	0.3	0.56
**9**	VA	Survival	120	80	100	74	100	100	100	100	2	2	2	1	3.2	3.6	3.0	3.0	0.3	0.27	0.29	0.18
**10**	VA	Survival	80	70	98	75	100	100	100	60	2	1.5	2.2	0.7	4.5	2.8	3.5	8.5	0.32	0.46	0.3	0.2
**11**	VV	Survival	89	83	72	70	100	100	100	21	2	1.5	1	0.1	8	5.2	5.6	7.9	0.39	0.4	0.38	0.52
**12**	VA	Survival	77	70	64	/	100	100	100	/	1.5	1	2	0	9	6.8	6.4	7.8	0.47	0.38	0.55	0.54
**13**	VV	Survival	88	83	76	58	100	100	100	/	1.5	1.5	1.6	0	9.6	6.2	6.8	8.2	0.41	0.4	0.46	0.84
**14**	VA	Survival	80	88	90	38	100	100	100	100	1.5	2	2	1	5	5.1	6	5	0.36	0.38	0.4	0.5
**15**	VV	Survival	80	77	76	70	100	100	80	/	1.2	0.8	0.7	/	10.4	10.8	10	/	0.42	0.4	0.5	/
**16**	VA	Survival	83	80	66	15	100	100	80	21	1.5	1.2	0.8	0	5.6	5.3	5.9	7.1	0.34	0.37	0.43	0.54
**17**	VA	Dead	85	80	82	75	100	100	100	100	2	1.8	1.8	1.9	4.3	4.1	4	3.7	0.32	0.30	0.29	0.27
**18**	VV	Survival	82	78	65	/	100	90	70	/	1.2	1.0	0.7	/	5.1	5.5	6.8	7.8	0.34	0.41	0.51	0.53

Oxygenation and haemodynamic parameters were monitored before and during ECMO. pH, PaO_2_/FiO_2_, PaCO_2_, MAP, the CI, LA, and the VIS were improved in both surviving VV-ECMO and VA-ECMO patients. Before ECMO initiation, compared with survivors, nonsurvivors were sicker, had a lower PaO_2_/FiO_2_ [49 (34.5, 62) vs. 63 (56, 71); *p* = 0.04], had a higher OI [41 (34.5, 62) vs. 30 (26.5, 35); *p* = 0.03], and had a higher VIS [30 (16.3, 80) vs. 100 (60, 142.5); *p* = 0.04] (Table 2).

Organ function at admission was recorded, and immune function was also monitored. No significant difference in organ function at admission was found, but nonsurvivors had less NK cell activity [0.7 (0.2, 3.1)% vs. 4.1 (3.3, 4.8)%, *P* =0.04] and a higher CD4+ T cell ratio [39.2 (31.1, 47.6)% vs. 28.6 (18.6, 34.2)%, *P* = 0.02] (Table 2.) One day after PICU admission, the ratio of CD4+ T cells was reduced in nonsurvivors compared with survivors [22.52 (18.09, 30.83)% vs. 29.64 (21.36, 33.01)%, *P* = 0.35].

### PICU management and ventilation settings

All ECMO patients received mechanical ventilation before ECMO. Pre-ECMO ventilation parameters were recorded, with a mean airway pressure of 22.5 (18.3–27.0) cmH_2_O, inspiratory pressure of 31 (27.8, 35) cmH_2_O, and positive end-expiratory pressure (PEEP) of 10.0 (6.0, 12.0) cmH_2_O. Among 18 patients, 83% required pre-ECMO vasopressors/inotropic drugs, with 27.8% (5/18) of patients receiving inhaled nitric oxide and 88.9% (16/18) receiving prone positioning (10-12 h/day).

When ECMO was established, the ventilatory settings were reduced as follows: 1) peak inspiratory pressure <25 cmH2O and PEEP 5-12 cmH2O; 2) respiration rate 15-25 breaths/min; and 3) FiO_2_ 0.3–0.5 [12]. On the basis of our centre’s experience, we suggest maintaining the tidal volume (Vt) above 3 ml/kg as much as possible to prevent alveolar collapse.

During ECMO support, 13 patients received continuous renal replacement therapy (CRRT) due to fluid imbalance and acute kidney injury (AKI). Patients with ARDS (11 cases) and radiologic bilateral dorsal exudation (5 cases) received prone positioning (10-12 h/day). Other therapies included neuromuscular blockade (18/18 cases), methylprednisolone at a dosage of 0.5-2 mg/kg.d for 5-7 days (18/18), intravenous immunoglobulin (18/18) at 400-500 mg/kg.d for the first three days, and parenteral nutrition (8/18). As oxygenation and haemodynamics improved with ECMO support, patients were weaned from vasoactives and inotropes. Patients were managed empirically with antibiotics if bacterial infection was suspected.

### Clinical outcomes and complications

**Figure 2:**
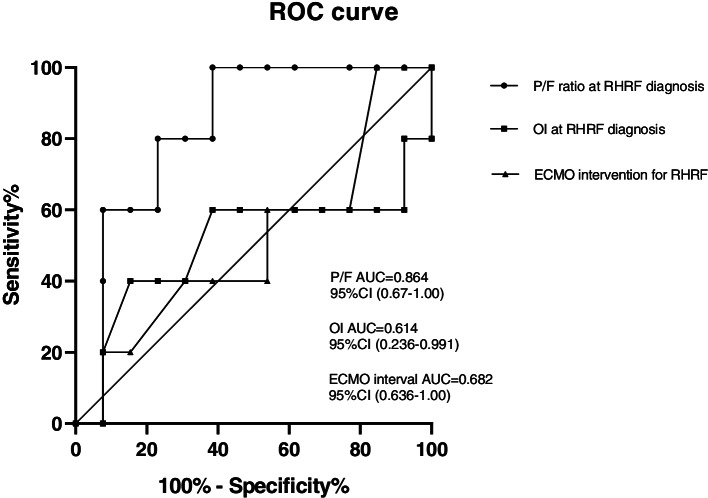
**Fig. 2** ROC curves for the prediction of poor outcomes **a** P/F ratio at RHRF diagnosis, **b** OI at RHRF diagnosis, **c** ECMO intervention for RHRF

The median ECMO duration was 196 (152, 309) h. The ECMO support duration was significantly longer in nonsurvivors than in survivors (244 [206.5, 478] hours vs. 166 [128.5, 269.5] hours, *P* = 0.03). The hospital survival rate was 72.2% among a total of 18 patients. Five patients died in the PICU: 1 from multiorgan failure, 2 from acute liver failure, 1 from intracranial haemorrhage, and 1 from refractory septic shock. We drew the ROC curve of the important parameters in nonsurvival patients. The AUC of P/F when RHRF diagnosed was 0.864, 95%CI (0.67-1.00); the AUC of OI when RHRF diagnosed was 0.614, 95%CI (0.236-0.991); The AUC of ECMO interval time from RHRF diagnosed was 0.682, 95%CI (0.36-1.00) (Fig. 2). PaO_2_/FiO_2_ <61 mmHg, OI >43 or hypoxic respiratory failure for more than 9 days before the initiation of ECMO are the cutoff points of ROC curves, which were associated with death.

Bleeding occurred in 8 patients, with 4 cases of cannulation-site bleeding, 3 cases of gastrointestinal bleeding, and 1 case of intracranial haemorrhage. There were 7 patients with severe thrombocytopenia (platelet count<50×10^9^/L). Nosocomial infections were detected in 4 patients. Of them, 2 patients had bloodstream infections caused by *Stenotrophomonas maltophilia*, 1 patient had infection caused by *Acinetobacter baumannii*, and 1 patient had infection caused by *Klebsiella pneumoniae*. *Acinetobacter baumannii* was isolated from bronchoalveolar lavage (BAL) fluid from 4 patients who developed ventilator-associated pneumonia. Those with nosocomial infections initially received empirical antibiotic therapy based on the pneumonia diagnosis, and antibiotic coverage was changed once ECMO was initiated. According to the data from CHINET, the antibiotics we chose covered carbapenem-resistant Acinetobacter, carbapenem-resistant *Klebsiella pneumoniae*, multidrug resistant *Pseudomonas aeruginosa*, and other carbapenem-resistant Enterobacteriaceae.

The ratio of patients complicated with ARDS or AKI was significantly higher in nonsurvivors than in survivors (*P*= 0.03, *P*= 0.001, respectively). There were no differences between survivors and nonsurvivors in terms of the ratio of complications of encephalopathy (*P*=0.09), liver dysfunction (*P*=0.06), shock (*P*=0.10), or gastrointestinal dysfunction (*P*=0.11). The median PICU stay (27 [19.5, 54] vs. 23 [16, 33], *P* = 0.31) and hospital stay were longer (48 [31.5, 71.5] vs. 28 [19, 34], *P*= 0.15) in nonsurvivors than in survivors, but the difference was not significant (Table 2).

## Discussion

We found that the hospital survival rate was 72.2% in children with severe Adv pneumonia who received ECMO support. We found that patients with RHRF with a PaO_2_/FiO_2_ <61 mmHg, an OI >43, or respiratory failure for more than 9 days had worse outcomes than their counterparts.

In paediatric patients, both PaO_2_/FiO_2_ and the OI were important variables to evaluate native lung function. To date, the ECMO mode and appropriate initial time of Adv pneumonia need further investigation. Indications for ECMO are currently based on data from adult patients. The OI had the best discriminatory value for mortality risk, with an area under the receiver operator curve of 0.747. An OI≥20, representative of severe paediatric ARDS, appears to be an appropriate cut-off point, as mortality increased from <23% in those with moderate paediatric ARDS to >40% in those with severe paediatric ARDS. Additionally, subjects with an OI>20 exhibited 40% mortality [10]. The current multicentre Extracorporeal Membranous Oxygenation for Severe ARDS study highlights the importance of time from intubation to ECMO [13]. Similar to a previous study [5], our research also showed that nonsurvivors exhibited a lower PaO_2_/FiO_2_ and higher OI before ECMO as well as longer times from intubation and RHRF confirmation to ECMO than survivors.

ECMO has been used as a salvage therapy for intractable hypoxia in patients with Adv pneumonia [3–6]. Based on the database of the ELSO registry, Ramanathan et al. [6] reported that patients with Adv pneumonia receiving ECMO had higher in-hospital mortality than those receiving ECMO for other infectious aetiologies. Among 321 paediatric severe Adv pneumonia patients treated with ECMO, 158 patients died, with an in-hospital mortality rate of 49.2% (158/321) [6]. According to our previous research, renal replacement therapy decreased in-hospital mortality in patients complicated with ARDS [14]; the mechanism involved cytokine clearance, and fluid management improved the outcome. Capillary leakage leads to impaired tissue perfusion and hypoxia, which trigger positive fluid balance and are associated with adverse outcomes [12]. In this group of patients, CRRT assisted with fluid management during the ECMO period. During the research period, due to early CRRT intervention, the conditions of 10 Adv pneumonia patients improved and they were able to avoid ECMO therapy. Moreover, we maintained the tidal volume above 3 ml/kg to prevent alveolar collapse during ECMO support. We also monitored dynamic pulmonary compliance during the ECMO period to prevent diffuse alveolar collapse as well as a complete lack of oxygenation in the native lung.

Severe Adv pneumonia has a high mortality rate and is associated with a higher risk of death in infants and young children. In the present study, 5 patients died. Viral virulence and immunocompetent/immune-compromised status have been well described in Adv infection [1, 15]. The innate immune response has been shown to play an important role in the host response to Adv infection [16]. Initial inflammation in Adv pneumonia presents as neutrophilic interstitial infiltrate, followed by the appearance of monocytes and lymphocytic infiltrate, and finally the release of cytokines such as IL-6 and IL-8 [17]. It has been suggested that Adv infection in an immunocompetent individual could result from the inhibition of cytokine production, suppression of T cell function, and inhibition of major histocompatibility complex (MHC) expression by virulent strains, such as HAdV-3 and HAdV-7 [18]. In our research, the nonsurvival group had a higher CD4+ T cell ratio than the surviving group at admission, and one week later, the CD4+ T cell ratio obviously decreased in the nonsurvival group. This could be explained by the following: first, neutrophil activation and accumulation in tissue caused the spread of inflammation; soon after, immune suppression occurred, worsening the outcome. A variety of modes of action have been proposed to explain the beneficial effects of the administration of intravenous immunoglobulin (IVIG), including its interactions with T cell function and antigen-presenting cell maturation/presentation and the general "calming" of inflammatory reactions. At doses higher than those required for replacement therapy, IVIG is being administered for the treatment of certain bacterial and viral infectious diseases [19]. It has been reported that some IVGg batches have significant activity against Adv, so patients are treated with IVIG [20]. Since cidofovir is not available, IVIG is used in many critically ill patients despite the lack of controlled trials.

There were several limitations to our study. First, only 18 patients received ECMO support in our retrospective analysis, which affected the power of the conclusion. Second, although all the surviving patients were followed regularly, long-term follow-up data are absent. To date, three of the patients developed complicated bronchiolitis obliterans, and one patient developed motor retardation, requiring rehabilitation therapy. The overall performance and cerebral performance data are incomplete. However, based on our experience, it was likely that ECMO support combined with other salvage therapies had beneficial effects on cardiopulmonary function in patients with severe Adv pneumonia.

## Conclusions

Our data provide insight into ECMO rescue in those with severe Adv pneumonia. Patients with a PaO_2_/FiO_2_ <61 mmHg or OI >43 and hypoxic respiratory failure for more than 9 days before the initiation of ECMO had worse outcomes.

## Supplementary Information

**Additional file 1.**

## Data Availability

The datasets used and/or analysed during the current study available from the corresponding author on reasonable request.

## Abbreviations

PICU: Pediatric intensive care unit
ECMO: Extracorporeal membrane oxygenation
Adv: Adenovirus
RHRF: Refractory hypoxic respiratory failure
AKI: Acute kidney injury
VIS: Vasoactive inotropic score
OI: Oxygen index
ARDS: Acute respiratory distress syndrome
ELSO: Extracorporeal Life Support Organization
CT: Computed tomography
CRRT: Continuous renal replacement therapy
mNGS: Metagenomic next-generation sequencing
CI: Cardiac index
LA: Lactic acid
ACT: Activated coagulation time
MAP: Mean arterial pressure
TBIL: Total bilirubin
sCr: Serum creatinine
IQR: Interquartile range
Vt: Tidal volume
IVIG: intravenous immunoglobulin
BALF: Bronchoalveolar lavage fluid
